# Spring Forward: Reproductive Phenology of the Holoparasite *Lathraea squamaria* (Orobanchaceae)

**DOI:** 10.1002/pei3.70101

**Published:** 2025-12-23

**Authors:** Yuliya Krasylenko, Luiza Teixeira‐Costa

**Affiliations:** ^1^ Department of Biotechnology, Faculty of Science Palacký University Olomouc Olomouc Czech Republic; ^2^ Plantentuin Meise Meise Belgium; ^3^ Meertens Instituut Amsterdam the Netherlands

**Keywords:** climate change, common toothwort, flowering, *Lathraea squamaria*, Orobanchaceae, parasitic plants, phenology, seed dispersal

## Abstract

*Lathraea* is a peculiar genus of holoparasitic plants in the Orobanchaceae. In addition to their unusual early development, plants in this genus remain below ground during most of their life cycle, deriving nutrients from the roots of various deciduous trees. In *Lathraea squamaria*, known as common toothwort, plants can persist underground for up to a decade before initiating flowering aboveground. To assess the effects of climate variability on the reproductive phenology and seed output of this species, we conducted a 14‐year population monitoring study. Our data show that the average onset of flower anthesis and seed dispersal have shifted −0.4 and −0.3 days/year over time, respectively. This resulted in these phenophases stating 5 days (anthesis) and 9 days (seed release) earlier in 2021 compared to 2007. Nevertheless, these phenological changes were not significantly correlated with local temperature and precipitation, suggesting that developmental timing in *L. squamaria* may be more influenced by host‐derived physiological cues. Indeed, early flowering has also been reported by one of the most common host species in the region, *Carpinus betulus* , the European hornbeam. Earlier flowering of common toothwort may also lead to temporal mismatches with pollinators, such as bumblebees. These findings underscore the importance of host–parasite synchrony in understanding the ecological resilience of holoparasitic plants under changing environmental conditions.

## Graphical abstract

*Lathraea squamaria* is a peculiar holoparasitic plant. Here, we show that over a 14‐year period, key stages in the reproductive phenology of this parasite have started significantly earlier. Most remarkably, initial dates of flower anthesis and seed release now occur five and nine days earlier, respectively, when compared to 2007.

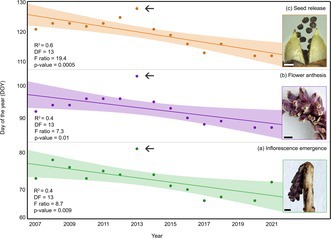

## 1 Introduction

*Lathraea* is a peculiar genus of parasitic plants. It is among the few genera in the Rhinantheae tribe composed exclusively of perennial species (Těšitel et al. 2010). At the same time, it differs from all other genera in the tribe by being the only one whose species are holoparasites, meaning they are entirely devoid of photosynthesis (Fu et al. 2017). Additionally, *Lathraea* is also different in terms of haustorium development. This organ, which promotes attachment, penetration, and connection to the host, differentiates from the apical root meristem soon after seed germination in nearly all holoparasites (Teixeira‐Costa and Davis 2021; Westwood et al. 2010). In *Lathraea*, however, multiple haustoria differentiate laterally from the roots in a process that can take up to 2 months (Atkinson and Atkinson 2020). Indeed, the overall growth of *Lathraea* plants below ground is characteristically slow, possibly due to their low sink strength in the uptake of host‐derived resources (Heide‐Jørgensen 2008).

**FIGURE 1:**
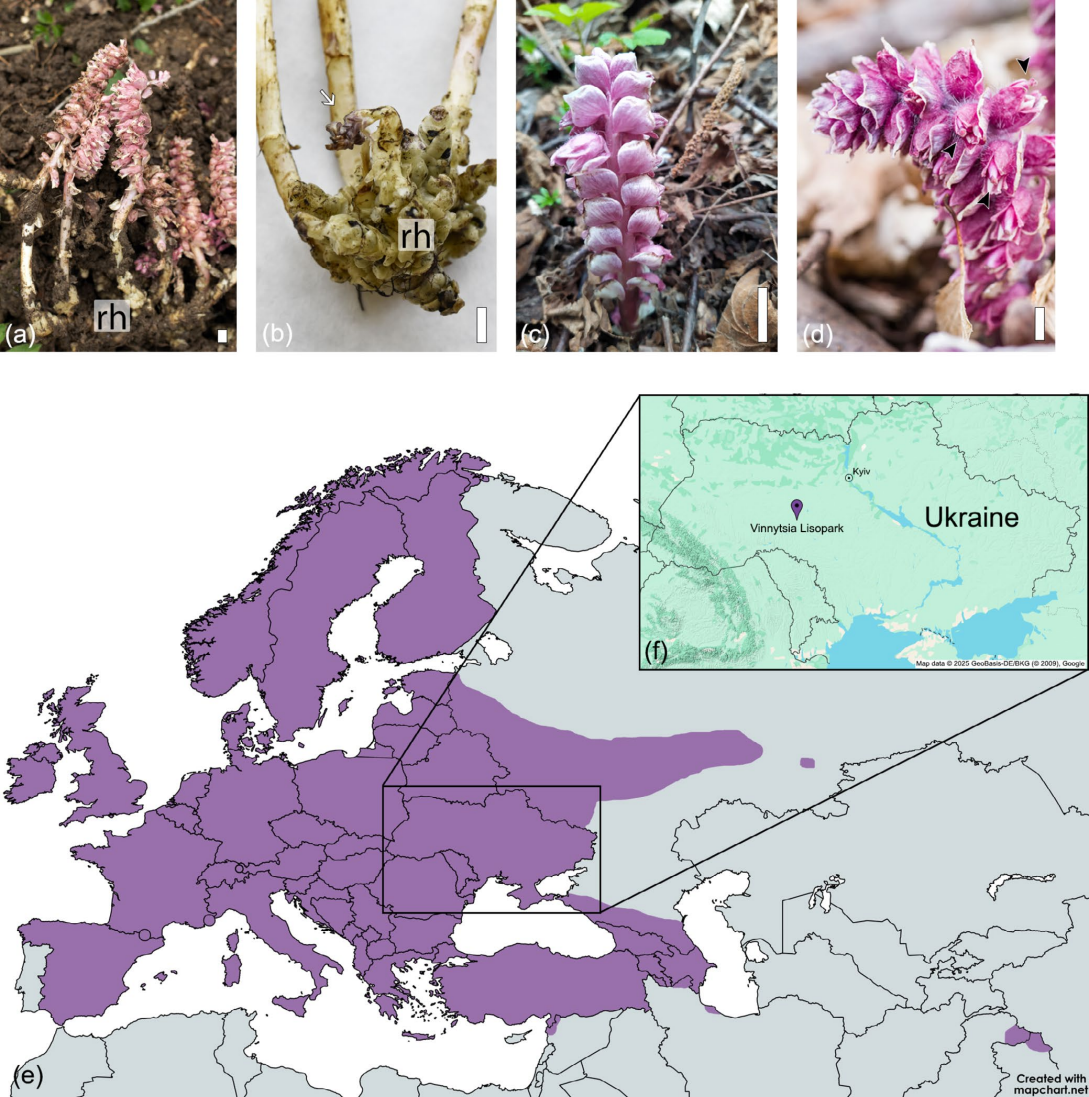
*Lathraea squamaria* general morphology and distribution range. (a) Long rhizome (rh) bearing multiple inflorescences. (b) Excavated rhizome (rh) bearing an inflorescence at an early developmental stage (arrow). (c) Mature inflorescence above ground. (d) Mature inflorescence bearing open flowers (arrowhead). (e) Distribution map of *L. squamaria* based on Hatt et al. (2024); image created with mapchart.net. (f) Map of Ukraine showing the location of the field site Vinnytsia Lisopark; image created with maps.co/gis. Scale bars: 1 cm.

This slow growth is most expressive in *Lathraea squamaria*, which has been reported to live exclusively below ground for about 10 years before the first inflorescences emerge through the soil surface (Kuijt 1969). After this long period, once *L. squamaria* plants reach reproductive age, multiple inflorescences emerge annually from the same underground rhizome (Figure 1a,b). In turn, each inflorescence bears dozens of flowers, light pink to bright purple in color (Figure 1c,d). Flowering is usually limited to the interval between March and May throughout most of the species’ extensive distribution range across Europe and central Asia (Figure 1e; Hatt et al. 2024). Considering this wide distribution, which is allied to a capacity of parasitizing an ample range of host trees, the apparent synchronicity in the flowering period of *L. squamaria* remains a puzzling question.

To date, few studies have analyzed the phenology of holoparasitic plants, especially concerning potential relationships with host phenology and environmental conditions. In the annual holoparasites *Orobanchae crenata* and *Phelipanche ramosa* (Orobanchaceae), short‐term monitoring (< 5 years) showed that climate conditions have a strong influence on parasite phenology (Arjona‐Berral et al. 1987; Moreau et al. 2016). Similar results were obtained in a study using herbarium specimens of *O. uniflora* collected over 100 years, which revealed reproductive phenology to be strongly influenced by spring temperatures (Park et al. 2019). Likewise, herbarium data for *L. squamaria* also suggest that, in areas of higher elevation, where temperatures tend to be lower, flowering phenology extends up to June–July (Hatt et al. 2024), raising the possibility that *L. squamaria* could be at least partially affected by climate variations. To address this question, we present here the results from a long‐term series of phenological observations for *L. squamaria* in western Europe.

## 2 Materials and Methods

A large population of *L. squamaria* was monitored weekly between late February and early June from 2007 to 2021 by Yuliya Krasylenko and her grandfather, Volodymyr Krasylenko at Vinnytsia Lisopark, west‐central Ukraine (49.239024, 28.420503; Figure 1f). The population occupied an area of approximately 35 m^2^ and grew around two large *Carpinus betulus* L. (Betulaceae) host trees. Considering multiple inflorescences emerge from a single rhizome and that determining individual plants requires excavating the soil, phenological observations focused on recording the onset date of three key phenophases: emergence of inflorescences above ground, flower anthesis, and seed release. To do so, the date of the first observation of each phenophase was recorded, which was typically visible in at least 5–10 inflorescences simultaneously.

To investigate the relationship between the onset date of each phenophase and time during the 14‐year period, a linear regression was fitted to the data. The same procedure was followed to analyze the influence of the local climate on phenological shifts. All analyses were carried out using JMP version 14 (SAS Institute Inc., Cary, NC, 1989–2025). Daily data on maximum, mean, and minimum temperature, as well as precipitation referring to the period from December 2006 to December 2021 at the Vinnytsia weather station were downloaded from the website of the European Climate Assessment and Dataset project (Klein Tank et al. 2002). Unfortunately, data relating to snow depth and snowmelt were either incomplete or not available for the analyzed period. From the available data, we obtained mean values for each climatic parameter corresponding to winter (December, January, and February) and spring (March, April, and May) periods. The focus on these two seasons was mostly due to the period of above‐ground phenological activity observed for *L. squamaria* (from March to early May), as well as the slow‐growing life cycle of this species.

## 3 Results and Discussion

**FIGURE 2:**
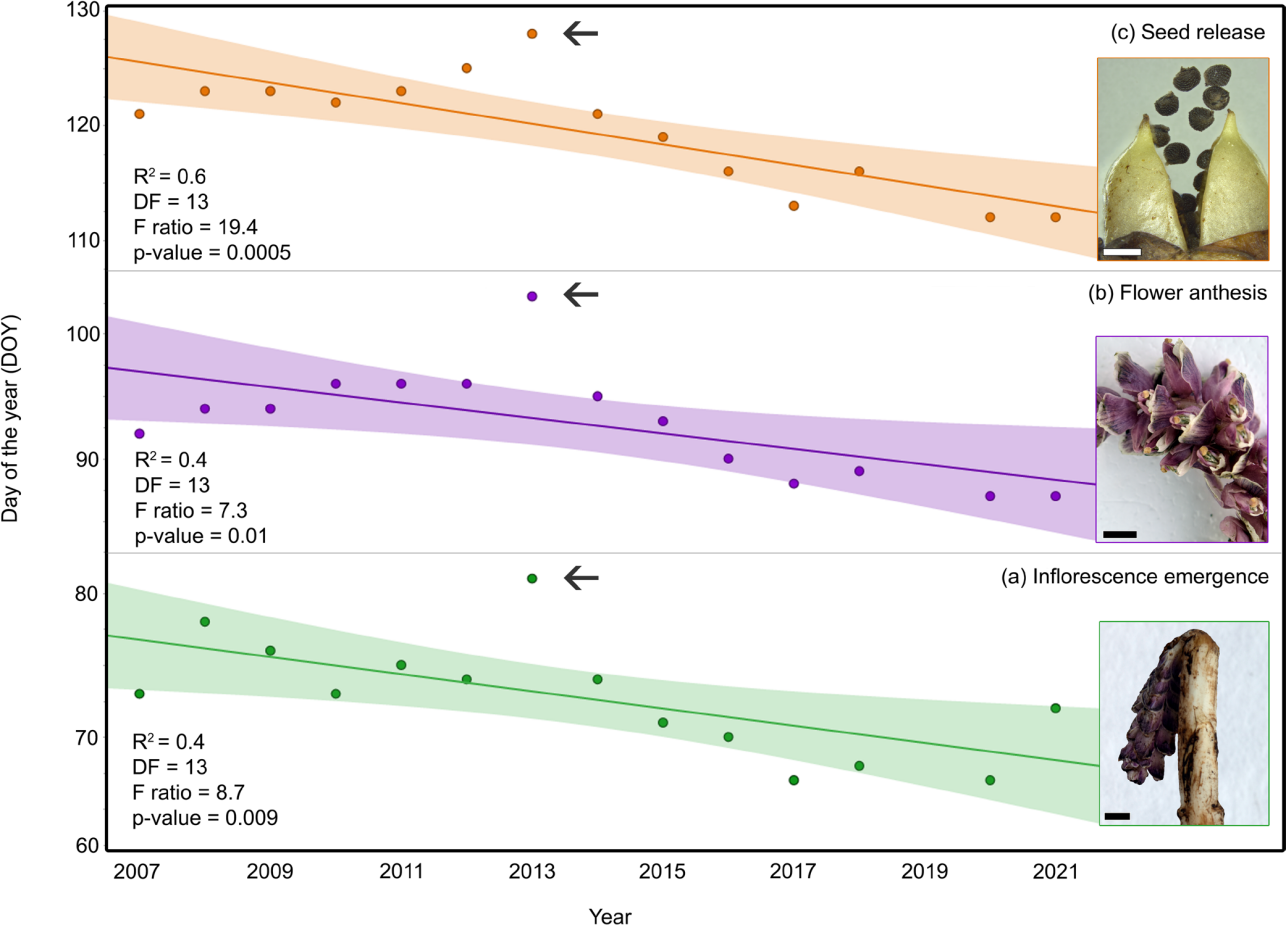
Progressively early onset of key reproductive phenophases in the lifecycle of *L. squamaria* between 2007 and 2021. (a) Inflorescence emergence from the soil. (b) Flower anthesis. (c) Seed release. Note the peak in 2013 (arrow) in which all phenophases were observed at their latest date throughout the entire period. White scale bar: 0.1 cm; black scale bars: 5 cm. Trend line, confidence interval, and summary statistics obtained through linear regression.

The above‐ground part of the life cycle in *L. squamaria*, which corresponds to its reproductive phenophases, takes place during a few weeks in early spring (late March–early May). In the 14‐year monitoring period from 2007 and 2021, there was a significant trend toward an earlier onset of inflorescence emergence (Figure 2a), flower anthesis (Figure 2b), and seed release (Figure 2c). While only a minor average shift was observed for inflorescence emergence (−0.08 days/year), the onset of flower anthesis shifted −0.4 days/year and occurred 5 days earlier in 2021 compared to 2007. Initial seed release dates shifted −0.3 days/year, occurring 9 days earlier in 2021 compared to 2007. The observed trend is particularly clear after 2013, when all three phenophases were observed at their latest recorded date for the entire period (Figure 2, arrow). In the same year, pollen traps located near Vinnytsia Lisopark captured a high concentration of pollen from the host species, *C. betulus* , with peak intensity during the last week of April (Rodinkova 2015). Considering the short span of the measurements, between 2009 and 2014, changes in atmospheric pollen concentration are more likely to indicate fluctuations in flowering activity, rather than profound changes in species composition. Therefore, as the pollen release peak recorded for *C. betulus* corresponds with the period between the onset of anthesis and the start of seed release observed for *L. squamaria* in 2013, we suggest a potential synchronicity between parasite and host regarding flowering at least in that specific year. Moreover, as high pollen production has been associated with abundant seed production in wind‐pollinated species (Pearse et al. 2016), the pollen data also suggest 2013 to have been a masting year for *C. betulus* . In this scenario, a high allocation of host resources into seed production could have depleted the parasite, causing a delay in its phenology.

Further evidence of phenological synchrony between parasite and host comes from long‐term monitoring of the host species. Although we were unable to locate additional phenological data for *C. betulus* growing in Ukraine, data from across Czechia show a strong trend toward progressively earlier flowering in *C. betulus* (Hájková et al. 2023). This trend matches our observations for *L. squamaria*, resulting in the onset of flowering in the host species continually fitting the progressively earlier period between anthesis and seed release recorded for the parasite. We hypothesize this phenological synchrony is mediated by a continued and prolonged exchange of hormones, sugars, and other signaling molecules between host and parasite throughout their perennial life cycles.

Moreover, no significant relationship was detected for *L. squamaria* between the onset of inflorescence emergence and average winter climatic variables, whether temperature (*p*‐values: maximum = 0.15; mean = 0.67; minimum = 0.68) or precipitation (*p*‐value = 0.43) According to the Köppen‐Geiger system, the climate of Vinnytsia is strongly characterized by snow accumulation during autumn and winter (November–March), which can create an insulating layer over the soil protecting the parasite from fluctuations in winter air temperatures (Kottek et al. 2006; Wilson et al. 2020). During spring, temperatures showed less variation, but again no significant relationship was detected between average spring temperatures and the start of either anthesis (*p*‐values: maximum = 0.68; mean = 0.82; minimum = 0.98), or seed release (*p*‐values: maximum = 0.98; mean = 0.84; minimum = 0.59). Spring precipitation was also not correlated with anthesis (*p*‐value = 0.83), nor seed release (*p*‐value = 0.66). These results agree with what has been reported for another perennial holoparasite *Langsdorffia hypogaea* (Balanophoraceae). In a region characterized by uneven rainfall, with ca. 90% of the annual precipitation occurring during the summer, parasite flowering and fruit maturation were not correlated with precipitation (da Silva Freitas et al. 2017).

Regarding interactions between *L. squamaria* and other species beyond its hosts, the advance in the parasite reproductive phenology can have an impact on a variety of small invertebrates that benefit from its nectar, which usually becomes available when few other plants are blooming (Rüther and Klotz 2009). This is most significant in relation to bumblebees (*Bombus* spp.), which are the most frequent pollinators (Hatt et al. 2024). The earlier onset of flower anthesis in *L. squamaria* could lead to nectar becoming available before the period of bumblebee activity, thus hindering pollination. This is especially important considering that the time span between the start of flower anthesis and the beginning of seed release has shortened on average 0.7 days/year over the observed period (*R* ^2^ = 0.6; *p*‐value = 0.02). This is likely due to an overall shorter flowering period, which could have a further impact on resource availability for pollinators. Alternatively, this change could reflect an acceleration of the seed development and maturation processes. No significant change was observed in the duration of the time between inflorescence emergence and anthesis.

## 4 Concluding Remarks

Altogether, our data show an advance in the reproductive phenology in *Lathraea squamaria*, with the onset of important phenophases, such as flower anthesis and seed release, occurring progressively earlier. In most plants, flower development is triggered not only by internal factors, such as plant age and gibberellin levels, but especially by external factors, including temperature and photoperiod, which are sensed by the leaves (Song et al. 2013). Due to the absence of photosynthesizing leaves and functional roots, the mechanism by which holoparasites could sense abiotic factors remains unclear. The absence of significant relationships between the phenology of *L. squamaria* and local climatic variables suggests that external factors might play a lesser role in regulating life cycle events compared to the internal influence of host‐derived signals. In this way, environmental conditions might influence *L. squamaria* in a manner that is indirect and mediated by the host *C. betulus* , whose reproductive phenology correlates with temperature variations in regions of Ukraine (Melnychenko et al. 2020) and Czechia (Hájková et al. 2023). For instance, the high temperatures recorded around Vinnytsia during July 2012 could have favored the occurrence of mast seeding in the host (Czeszczewik et al. 2020), indirectly affecting parasite phenology via resource redistribution.

In this context, host identity becomes an important factor, and future research should explore the phenology of this peculiar parasite in association with other host species. This is especially relevant considering that, in Ukraine, the host *C. betulus* is at the edge of its geographical range, making it more exposed to extreme climatic events (Tytar 2023). Observations from other parasite–host associations reinforce the importance of host identity for parasite phenology. Recent results for the endoparasite *Cytinus hypocistis* (Cytinaceae), which is also devoid of photosynthetic activity, show that its flowering phenophase overlaps with only one out of three host species (de Vega et al. 2024). Moreover, although the reproductive phenology of *P. ramosa* was asynchronous with the host at natural light, greater synchronicity between parasite and host was observed under shaded conditions (Moreau et al. 2016). This observation highlights the potential impact of microclimatic conditions on parasitic plant phenology and development. In the annual holoparasite *Epifagus virginiana* (Orobanchaceae), for instance, variation in soil temperature, moisture, and pH affected a range of morphofunctional traits of the parasite tuber (Andrés‐Hernández et al. 2024). In geophytes, which also develop mostly below ground and flower upon emergence, warmer soil temperatures can restrict sexual reproduction and vegetative growth, especially for species inhabiting temperate regions (Sunmonu and Kudo 2015).

Finally, it is also noteworthy that *L. squamaria* plays a multifaceted role in forest ecosystems, providing resources not only to its pollinators above ground, but also to indirectly associated species such as other small insects and neighboring plants below ground (Heide‐Jørgensen 2014). In this way, the presence of *L. squamaria* often serves as an indicator of ecosystem diversity and stability in forests across Europe (Rüther and Klotz 2009). Therefore, further research into how the phenology of above and below ground developmental stages is integrated, and how it might impact other species of plants and animals should also be a prime target for future investigations.

## Funding

This work was supported by Koninklijke Nederlandse Akademie van Wetenschappen; Carlsberg Ukraine, Science Battle individual grant.

## Conflicts of Interest

The authors declare no conflicts of interest.

## Supporting information


**Data S1:** Year‐to‐year variation in the onset of major reproductive phenophases (inflorescence emergence, flowering, and seed release) of *Lathraea squamaria* monitored from 2007 to 2021. DOY, day of year.

## Data Availability

Raw data related to phenological observations is available as Supporting Information.
